# Home-Based versus Hospital-Based Rehabilitation Program after Total Knee Replacement

**DOI:** 10.1155/2015/450421

**Published:** 2015-04-16

**Authors:** Remedios López-Liria, David Padilla-Góngora, Daniel Catalan-Matamoros, Patricia Rocamora-Pérez, Sagrario Pérez-de la Cruz, Manuel Fernández-Sánchez

**Affiliations:** ^1^Department of Nursing, Physiotherapy and Medicine, University of Almería, La Cañada de San Urbano, 04120 Almería, Spain; ^2^Department of Psychology, University of Almería, La Cañada de San Urbano, 04120 Almería, Spain; ^3^Health Communication, University Carlos III of Madrid, Getafe, 28903 Madrid, Spain

## Abstract

*Objectives*. To compare home-based rehabilitation with the standard hospital rehabilitation in terms of improving knee joint mobility and recovery of muscle strength and function in patients after a total knee replacement. *Materials and Methods*. A non-randomised controlled trial was conducted. Seventy-eight patients with a prosthetic knee were included in the study and allocated to either a home-based or hospital-based rehabilitation programme. Treatment included various exercises to restore strength and joint mobility and to improve patients' functional capacity. The primary outcome of the trial was the treatment effectiveness measured by the Western Ontario and McMaster Universities Osteoarthritis Index (WOMAC). *Results*. The groups did not significantly differ in the leg side (right/left) or clinical characteristics (*P* > 0.05). After the intervention, both groups showed significant improvements (*P* < 0.001) from the baseline values in the level of pain (visual analogue scale), the range of flexion-extension motion and muscle strength, disability (Barthel and WOMAC indices), balance, and walking. *Conclusions*. This study reveals that the rehabilitation treatments offered either at home or in hospital settings are equally effective.

## 1. Introduction

The success of total knee arthroplasty in reducing pain, restoring physical functions, and improving the quality of life of people with severe osteoarthritis of the knee is now well established [1–5]. Pain relief and functional restoration, specifically the ability to achieve an adequate range of motion (ROM), including stair climbing and walking, are important goals of rehabilitation [5, 6]. Various authors have conducted systematic reviews [7] and meta-analyses [8] and reported that intensive functional rehabilitation during the subacute recovery period after primary total knee arthroplasty improved short- and mid-term functional capacity, pain intensity, gait velocity, cadence and stride length, and quality of life [9–11].

Traditionally, physical therapy has been a routine component of patient rehabilitation following knee replacement [12]. In Spain, patients are usually sent home 4 or 5 days after the replacement and called later on to receive outpatient rehabilitation at the hospital. However, in recent years this practice is being modified. Various alternatives are emerging for patients to receive physical therapy treatments, such as at public health centres or at patient's home.

In Spain, home-based rehabilitation support or rehabilitation in the home (RITH) has been a usual practice for many years (patients have had to hire physiotherapists privately to attend to them at home). However, the use of RITH as a welfare method of health departments in the public administration is relatively recent [13]. Catalonia was the first community to offer RITH as a public service in 1991, with no direct cost to the user. This was created to facilitate the treatment of patients who could not come to the hospital due to comorbidities or architectural barriers in their homes (i.e., stairs). In Andalusia (the Andalusian Health Service), a mobile rehabilitation and physical therapy team (rehabilitation physician, physiotherapist, and occupational therapist) started operating in 2002 as the exclusive provider of this public home service [14].

In Europe, the trend has been to shorten in-hospital convalescence and to increase the reliance on community-based therapy for rapid functional recovery. For patients to be discharged home, some health professionals consider personal/psychosocial factors such as patients' goals and their social support [15].

Due to the increase in total joint replacements worldwide, further investigation is needed on how to meet the rehabilitation needs of people undergoing this type of surgery. There is an increasing emphasis on achieving cost-effectiveness in care; and health systems are currently under strong economic pressure. Therefore, reducing the length of hospital stay has become a priority [16, 17].

There are several general reviews in the literature on rehabilitation following knee arthroplasty [9], and considerable international research about RITH has been done over the past 10 years [18]. Theoretical models and qualitative and quantitative studies all acknowledge the influence of the environment on functioning [19, 20]. When the activities of daily living (ADL) are carried out in the home setting, the situation is more meaningful to a patient than artificial simulations in a clinical setting [19].

Several authors [21, 22] have conducted studies to compare rehabilitation programs in hospital and at home after a total knee replacement (TKR) and have found no group differences in the functional outcomes. Kuisma [23] and Dow [24] emphasised the risk of early discharge of patients from the hospital and its impact on the families. However, Kauppila's [1] findings were in favour of RITH versus inpatient rehabilitation, even after early hospital discharge. Mahomed et al. [25] showed a significant reduction in the cost of care by using home-based rehabilitation programs, without compromising its quality.

In Spain, it has not yet been determined whether RITH accelerates patient improvement or the outcome is independent of the environment. There is still conflicting evidence on the benefits of RITH versus outpatient hospital rehabilitation after a total joint replacement [9].

The aim of this study was to compare RITH with standard outpatient hospital rehabilitation in terms of improving knee joint ROM, muscle strength, and functional recovery of patients with a prosthetic knee.

## 2. Materials and Methods

**Figure 1:**
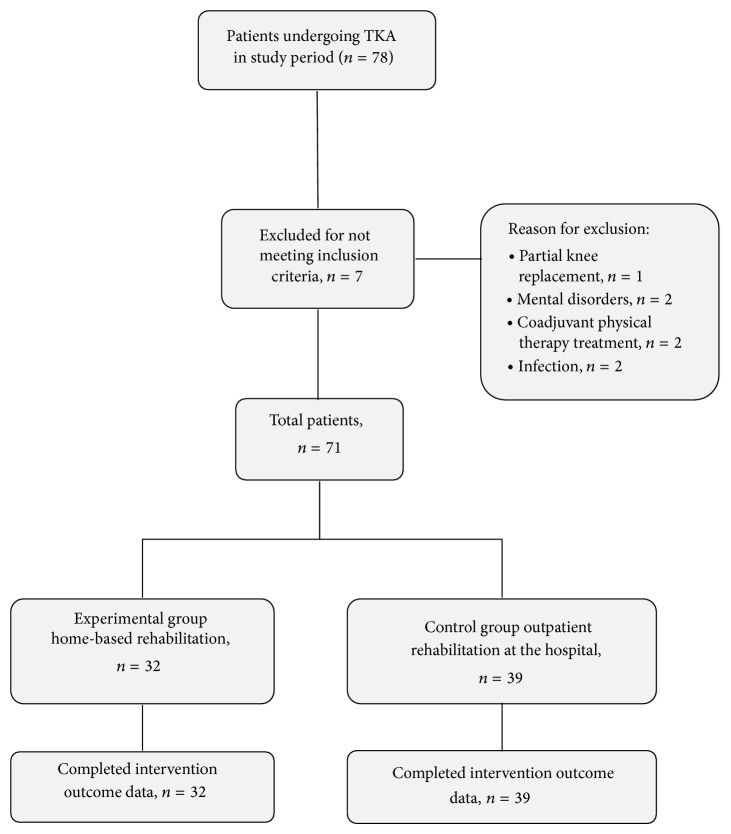
Flow of the patients through the study.

This study was based on a nonrandomised controlled trial. After completing their postoperative period, 78 patients from the Traumatology Unit of the Torrecárdenas Hospital Complex (Almería) were recruited to participate in the trial comparing standard and home-rehabilitation (Figure 1). The study was approved by the Human Research and Ethics Committee of the participating Health Service. All patients provided written informed consent in accordance with the Helsinki Declaration. The treatment condition (hospital/home) was selected by rehabilitation physician Dr. I. G., mainly on the basis of the need for assistance in ADL, the characteristics of patients' homes (architectural barriers), and the availability (or lack) of social and family support, in accordance with the Rehabilitation Method Guidelines of the Regional Ministry of Primary Care in Andalusia [26].

The experimental group consisted of patients who received RITH, and the control group included outpatients who were treated in the Rehabilitation and Physical Therapy Department of the hospital (usual care).

The number of patients required to achieve statistical significance was determined by power analysis. We assumed that a difference of more than 5° of knee flexion mobility (SD 8°) at the end of the treatment would be clinically relevant. With an alpha of 0.05 and a power of 80%, we needed 28 patients per group to prove this.

### 2.1. Inclusion and Exclusion Criteria

The inclusion criteria were as follows: a total knee replacement operation, over 60 years of age, and voluntary participation in the study. The following exclusion criteria were used: major postoperative complications (hemarthrosis, a fracture or infection of the operated knee joint and deep vein thrombosis), psychiatric diagnosis, concurrent physical therapy treatment at a different institution, and the existence of a terminal disease with a life expectancy of less than 6 months. Of the 78 initially selected patients, 71 took part in the study. The final sample was comprised of 32 patients in the experimental group (RITH) and 39 patients in the control group (Figure 1).

### 2.2. Outcome Measures

All subjects were assessed by an external physiotherapist who did not participate in the patients' treatment (R. L.), on the 5th postoperative day. The assessment included basic demographic data (age, sex), the knee affected (right/left), the presence of comorbidities (diabetes, obesity (body mass index ≥ 30), and arterial hypertension), the receipt of pharmacological treatment prior to the intervention, and participation in ADL (using the Barthel Index to determine the degree of independence) [27, 28].

The primary measuring tool for the outcome of the study was the Western Ontario and McMaster Universities Osteoarthritis Index (WOMAC) used to assess pain (5 items), stiffness (2 items), and function (17 items) [29]. High WOMAC scores indicate poorer function and more severe pain and stiffness levels. The descriptors range from 0 (*no problems with pain, functional activities, and stiffness*) to 4 (*extreme pain, difficulty, and stiffness*).

We also measured participants' joint-specific pain using a 0–10 cm visual analogue scale (VAS). The patients rated the highest intensity of pain experienced in their operated knee in the previous 24 hours, with the scores ranging from 0 (*no pain*) to 10 (*worst imaginable pain*). The following measurements were also taken: passive knee ROM measured with a goniometer (expressed in degrees), flexion assessed while sitting and extension assessed while in supine position [30], muscle strength (quadriceps and hamstrings), and functional ambulation. This latter was assessed through gait and balance observation using the 22-item Tinetti test [31], which was divided into two subscales, Static Balance and Balance while Walking, each scored on a 3-point ordinal scale (0 =* abnormal*, 1 =* adapted,* and 2 =* normal*).

At the end of treatment, the same physiotherapist (R. L.) assessed the same baseline variables, as well as the duration of hospital stay (in days), the number of rehabilitation sessions received by each patient, the complications detected, and the readmission cases during the rehabilitation period.

### 2.3. Intervention

On the second day of the postoperative period and after having had an X-ray, all the patients (experimental and control groups) received prophylactic antibiotics and prophylaxis against deep vein thrombosis. The patients started physical therapy at the Traumatology Unit, according to the previously established and standardized care guidelines for a total joint replacement. This included health education for patients and families as well as the following: postural treatment; passive kinesiotherapy for the lower limb and cryotherapy for one hour thrice a day (once after passive mobilisation); muscle strengthening exercises (isometric exercises for quadriceps) and stretching of quadriceps and hamstrings; active flexion-extension exercises for the knee and ankle with no resistance; flexion-extension while sitting; isotonic exercises; and facilitation of position changes from lying to sitting, sitting to standing (transfer from bed to chair)*¸* and standing and walking (short distances) [32]. Depending on their progress, this training on transfers was gradually carried out and the patients started walking with a walker between the third and the fourth days of the postoperative period. They were provided with instructions on exercises (passive, active-assisted and active flexion-extension bed and chair exercises and gait training, beginning with assisted walking), which they were recommended to perform daily after discharge.

The participants assigned to RITH were referred to their respective community health centre and included in an early intervention program that ensured that each patient was assessed at home by a rehabilitation physician approximately 72 hours after the discharge (the average delay in starting physical therapy at home was 13 days). The principal aim of the RITH program was to improve patients' quality of life and functional capacity by improving strength, increasing joint mobility, improving endurance, and motivating the patients to carry out a regular exercise program. The patients assigned to RITH spent an average of 28.59 minutes (SD 7.53) per treatment session with a physiotherapist. The functional exercises included transfers, gait training, and stair climbing [1, 25]. Muscle work was intensified daily and increased as the patients progressively adapted to ADL.

In the case of the control group, the hospital rehabilitation appointment was made before the patient was discharged (the average delay in starting outpatient physical therapy treatment at the hospital was 17 days). In the hospital, a supervised exercise program was developed by physiotherapists and it included various exercises to restore strength and joint mobility, such as walking without crutches, further joint mobility exercises, and strengthening exercises (isometric and dynamic) without external loads [1], with an average duration of 78.85 minutes per session (SD 14.48).

The patients were discharged from the RITH or hospital rehabilitation programs when they achieved sufficient functional improvement (at least 90° knee flexion; a score higher than 3 on the scale that measured quadriceps and hamstrings strength; independent walking).

### 2.4. Data Analysis

The results were analysed using the SPSS software Version 18.0 for Windows (SPSS Inc., Chicago, IL, USA). After conducting descriptive analysis of the variables (means and standard deviations), we performed baseline comparisons of the two treatment groups to determine whether they were equivalent on the measured variables. Next, within-group comparisons of pre- and postintervention scores were performed using *t*-tests, and between-group comparisons of change scores on all outcome measures were performed using *t*-tests, with a confidence interval of 95% (*P* = 0.05).

## 3. Results

The mean age of the patients was 71.27 years (SD = 6.52 years), and approximately two-thirds were women (70.40 percent). The groups did not differ significantly in the leg side (right/left) or in clinical characteristics. The mean length of stay in the hospital was 6.63 days (SD = 1.91) in the RITH group and 6.59 days (SD = 1.58) in the control group, with no statistically significant differences (*t* = 0.85, *P* = 0.933). A summary of patients' clinical characteristics for the study population is presented in Table 1.

In the RITH group, 65.60 percent of the patients were treated three days per week (Monday, Wednesday, and Friday), and 34.40 percent were treated two days per week (Tuesday and Thursday). In the control group, 67.60 percent of the patients were treated two days per week, and the remaining 32.40 percent were treated three days per week. This variable yielded statistically significant differences (*χ* ^2^ = 26.67, *P* < 0.001). The average number of physical therapy sessions for the RITH patients was 15.66 (SD = 7.11), whereas the average number of physical therapy sessions in the hospital was 14.74 (SD = 3.22), with no statistical group differences (*t* = 0.717, *P* = 0.506).

Initially, the patients in both groups had considerable pain, restricted range of joint motion, and functional disability, as can be seen from the scores obtained via various assessment questionnaires (Table 2). After rehabilitation, both groups showed significant improvements from the baseline values in pain VAS, range of flexion-extension motion and muscle strength, disability (Barthel and WOMAC), balance, and walking (Table 2). The final outcome of the process was a discharge due to improvement in 100 percent of cases in both groups. The extent of improvement after physical therapy was similar in both groups on most outcome measures; however, there was a significant difference in the level of improvement in knee extension ROM (*P* = 0.027) and muscle strength in the knee affected (*P* = 0.035) in favour of the hospital group (Table 3).

The average number of visits to the emergency ward due to complications during recovery was 0.13 (SD = 0.421) in the RITH group and 0.10 (SD = 0.307) in the control group. There were no statistically significant differences in the number of emergency visits (*t* = 0.259, *P* = 0.796) or in hospital readmissions (*t* = −0.905, *P* = 0.369).

## 4. Discussion

This study revealed that the RITH and hospital-based programs were largely comparable, with the exception of a better improvement in knee extension and strength in the hospital-based group. To understand the reason for this difference, we must consider that the initial assessment already included differences, since the hospital patients started their rehabilitation treatment later, which could explain their greater muscle atrophy and lesser extension. However, in the final assessment the muscle strength and extension achieved were similar for both groups. This was due to not only the treatment received, but also the normalisation of their activity over time. This elapsed time was greater for the hospital patients because although the number of sessions was the same for both groups, their frequency was different (2 sessions a week in hospital versus 3 sessions a week at home) so that the total elapsed time for the hospital patients was greater.

However, the functional capacities were not different between the groups following the intervention. This research confirms the data obtained by other authors such as Mitchell et al. [21], who conducted a study with similar experimental and control group characteristics and outcome variables. They found no statistically significant differences in the results based on the WOMAC scale, physical therapy satisfaction, or quality of life (the home group reported that they would prefer any future therapy to be home-based, having had positive experience with the home-based therapy program).

Mahomed et al. [25] have shown that the cost of care following a total hip or knee replacement can be significantly reduced by using home-based rehabilitation programs, without compromising the quality of care, as evidenced by comparable functional outcomes and patient satisfaction rates for up to one year after surgery. The findings from this study certainly support the concept of RITH following uncomplicated, primary total hip or knee joint replacement [25].

Kauppila's [1] findings favour RITH versus inpatient rehabilitation, even after an early hospital discharge. When the results of RITH and inpatient rehabilitation following primary total hip or knee replacement were compared using validated outcome measures, no differences in clinical outcomes were found 3 and 12 months after surgery. Both treatment groups achieved similar improvements in pain and function, and the percentage of complications was comparable to that in other studies [33–36]. Our research supports Kauppila's conclusions, even though there were no statistically significant differences in treatment effects between the groups [1]. We suggest that patients and caregivers may require a combination of hospital rehabilitation and home-based rehabilitation to meet their needs and preferences at each phase of the continuous rehabilitation process [37].

In a nonrandomised Australian trial, Tribe et al. [22] compared the functional outcomes for patients who had received either home-based or inpatient rehabilitation after undergoing total hip and knee replacements for primary osteoarthritis. There were no group differences in functional outcomes in a one-year follow-up assessment.

In general, staff and patients considered the home as providing adequate conditions for individualised, goal-directed therapy for medically stable rehabilitation patients [37]. RITH was viewed as advantageous for patients who could not access hospital-based therapy, granting them more control over the direction, timing, and duration of their therapy.

Research such as that published by Dow in 2004 [24] illustrates the personal impact on families of receiving a patient at home after early discharge. This causes concerns and increases expenses that are invisible to health and social workers. Other authors, such as Kuisma [23], highlight the risk of discharging a patient from hospital without any professional supervision.

In view of the suggestion that RITH therapy may enhance the therapeutic relationship, patient motivation, and patient and family involvement in rehabilitation, it can be hypothesised that RITH will also enhance performance and rehabilitation outcomes [19, 37]. Coordinating the rehabilitation process across disciplines and focusing on enhancement of patient participation may help improve the consistency and quality of patient engagement.

This study has several strengths. The population was representative of the current clinical practice; there was no manipulation of patient's treatment assignment or the techniques used and we used validated scales and considered that the procedure did not significantly affect the results. At the same time, we should admit some differences in the intervention content, potentially influencing our findings, in addition to the treatment setting, such as the frequency of treatment sessions, different therapists across settings, and differences in programme content between the groups, as described in the intervention section. It also has some other limitations. The allocation of patients to groups was not randomised and, therefore, our results cannot necessarily be generalised to the wider population of people with TKR in Spain.

Growing evidence shows that RITH programmes are at least as good as inpatient postoperative rehabilitation programs in terms of achieving functional outcomes for patients. In terms of future research directions, determining the most appropriate setting for community rehabilitation and the impact of the rehabilitation setting on outcomes is a key priority, given the diversity of settings. It is also an important element for treatment optimisation and diversification of resources for the people most in need.

The vulnerable elderly population, whose functionality is compromised and whose access to and use of resources are limited, needs personalised care with coordinated resources and highly capable professionals who can provide solutions to their problems. Home-based care requires a specific assessment of recipients' satisfaction, which is hardly comparable to the provision of services in other environments [38]. It is essential to have a network of primary care and home-care programs that can establish the entire preventive, educational, and response potential for the health needs of this population. This requires the support of closely coordinated specialised services. These prevention measures can help reduce the impact of disease by preventing exacerbations of chronic conditions, consequences of immobility, and caregiver overload.

## 5. Conclusion

This study found the RITH and hospital-based programs to be largely comparable. We observed positive changes in patients' conditions in all the areas assessed including pain, functionality, walking, and balance. We can therefore confirm that the physical therapy treatments provided at home and in the hospital settings were equally effective. We only found significant differences in the initiation of treatment after the intervention (home-based treatment started earlier), in treatment frequency (higher for home-based treatment), in the average duration of sessions (longer in the hospital), and in the improvement of knee extension and strength (greater in the hospital-based group).

## Figures and Tables

**Table 1:** Comparison of preoperative patients' clinical characteristics.

Assessments	Total patients (*N* = 71)	RITH (*n* = 32)	Hospital-based rehabilitation (*n* = 39)	Student's *t* or *χ* ^2^
	M (SD), %	M (SD), %	M (SD), %	*P* value
Age (mean ± SD)	71.27 (6.52)	72.78 (7.61)	70 (5.22)	0.093
Sex (%)				
Women	70.40%	75%	66.70%	0.614
Men	29.60%	25%	33.30%	
Basal				
Barthel index	94.08 (5.68)	93.44 (6.77)	94.62 (4.64)	0.407
Diabetes (%)	23.90%	28.10%	20.50%	0 .214
Obesity (%)	35.20%	34.40%	35.90%	1.000
Hypertension (%)	66.20%	62.50%	69.20%	0.731
Bilateral				
Prosthesis (%)	29.60%	28.10%	30.80%	1.000
Leg (%)				
Right	50.70%	43.80%	56.40%	0.410
Left	49.30%	56.20%	43.60%	
Pharmacological treatment (%)	97.20%	96.90%	97.40%	1.000

Note: the *P* value represents differences between the RITH group and the control group through comparison of independent samples with Student's *t*- or *χ* ^2^ test for categorical variables; *P* < 0.05.

**Table 2:** Related samples test. Pretest versus posttest, RITH group and control group.

Tests	Related samples test			Related samples test		
	Pretest versus posttest			Pretest versus posttest		
	RITH			Hospital-based rehabilitation		
	1st assessment M (SD)	2nd assessment M (SD)	*P* value	1st assessment M (SD)	2nd assessment M (SD)	*P* value
Pain (VAS: 0–10)	7.03 (2.27)	2.75 (2.39)	<0.001	7.13 (1.96)	2.38 (2.40)	<0.001
Knee range of motion						
Flexion	87.03 (9.14)	100 (7.29)	<0.001	85.95 (7.54)	99.67 (7.95)	<0.001
Extension	−5.63 (10.60)	−0.31 (1.23)	<0.001	−10.90 (4.42)	−0.72 (2.87)	<0.001
Muscle strength	3.03 (0.69)	4.59 (0.49)	<0.001	2.59 (0.54)	4.51 (0.50)	<0.001
Barthel index [27]	55.94 (8.74)	97.19 (4.00)	<0.001	54.49 (6.46)	99.10 (6.65)	<0.001
WOMAC [29]						
Pain (0–20)	12.34 (3.60)	2.50 (2.96)	<0.001	13.64 (3.05)	3.56 (2.87)	<0.001
Stiffness (0–8)	4.13 (1.60)	1.16 (1.16)	<0.001	4.90 (1.42)	1.33 (0.92)	<0.001
Physical function (0–68)	51.81 (6.37)	13.19 (7.50)	<0.001	55.46 (6.08)	15.36 (8.85)	<0.001
Tinetti test [31]						
Gait (12)	5.37 (1.92)	11.77 (0.77)	<0.001	6.11 (2.43)	11.82 (0.45)	<0.001
Balance (16)	9.17 (2.47)	15.60 (1.19)	<0.001	9.79 (1.93)	15.97 (0.16)	<0.001
Global Tinetti	14.53 (3.97)	27.37 (1.93)	<0.001	15.89 (3.81)	27.79 (0.52)	<0.001

**Table 3:** Differences between the RITH and control control group.

Tests		RITH		Hospital-based rehabilitation		Differences between groups
		M	SD	M	SD	*P* value
Pain (VAS: 0–10)	1st assessment	7.03	2.27	7.03	1.96	0.848
	2nd assessment	2.75	2.39	2.38	2.40	0.525
	Effect	−4.28	2.39	−4.74	2.34	0.416

Knee range of motion						
Flexion	1st assessment	87.03	9.14	85.95	7.54	0.586
	2nd assessment	100.00	7.29	99.67	7.95	0.856
	Effect	12.97	8.31	13.72	8.77	0.715
Extension	1st assessment	−5.63	10.60	−10.90	4.42	0.022^*^
	2nd assessment	−0.31	1.23	−0.72	2.87	0.429
	Effect	5.31	11.14	10.18	4.86	0.027^*^

Muscle strength	1st assessment	3.03	0.69	2.59	0.54	0.004^*^
	2nd assessment	4.59	0.49	4.51	0.50	0.502
	Effect	1.56	0.75	1.92	0.62	0.035^*^

Barthel index [27]	1st assessment	55.94	8.74	54.49	6.46	0.439
	2nd assessment	97.19	4.00	99.10	4.11	0.052
	Effect	41.25	8.61	44.62	7.19	0.083

WOMAC [29]						
Pain (0–25)	1st assessment	12.34	3.60	13.64	3.05	0.105
	2nd assessment	2.50	2.96	3.56	2.82	0.127
	Effect	−9.84	3.53	−10.08	2.80	0.757
Stiffness (0–10)	1st assessment	4.13	1.60	4.90	1.42	0.035^*^
	2nd assessment	1.16	1.16	1.33	0.92	0.478
	Effect	−2.97	1.53	−3.56	1.37	0.890
Physical function (0–85)	1st assessment	51.88	6.37	55.46	6.08	0.018^*^
	2nd assessment	13.19	7.50	15.36	8.85	0.278
	Effect	−38.69	7.96	−40.10	8.87	0.486

Tinetti test [31]						
Gait (12)	1st assessment	5.37	1.92	6.11	2.43	0.179
	2nd assessment	11.77	0.77	11.82	0.45	0.745
	Effect	6.40	2.25	5.71	2.44	0.237
Balance (16)	1st assessment	9.17	2.47	9.79	1.93	0.248
	2nd assessment	15.60	1.19	15.97	0.16	0.099
	Effect	6.43	2.81	6.18	1.92	0.667
Global Tinetti	1st assessment	14.53	3.97	15.89	3.81	0.156
	2nd assessment	27.37	1.93	27.79	0.52	0.240
	Effect	12.83	4.70	11.89	3.85	0.369

^*^Means that the results were statistically significant, with a confidence interval of 95% (*P* = 0.05).
